# Early-Onset Endocrine Disruptor–Induced Prostatitis in the Rat

**DOI:** 10.1289/ehp.11239

**Published:** 2008-03-26

**Authors:** Prue A. Cowin, Paul Foster, John Pedersen, Shelley Hedwards, Stephen J. McPherson, Gail P. Risbridger

**Affiliations:** 1 Centre for Urological Research, Monash Institute of Medical Research, Monash University, Clayton, Victoria, Australia; 2 National Institute of Environmental Health Sciences, National Institutes of Health, Department of Health and Human Services, Research Triangle Park, North Carolina, USA; 3 Tissupath Laboratories, Hawthorn, Victoria, Australia

**Keywords:** antiandrogen, endocrine disruptors, inflammation, prostate, prostatitis, vinclozolin

## Abstract

**Background:**

Androgens are critical for specifying prostate development, with the fetal prostate sensitive to altered hormone levels and endocrine-disrupting chemicals (EDCs) that exhibit estrogenic or antiandrogenic properties. Prostatic inflammation (prostatitis) affects 9% of men of all ages, and > 90% of cases are of unknown etiology.

**Objectives:**

In this study we aimed to evaluate effects of *in utero* exposure to the antiandrogenic EDC vinclozolin, during the period of male reproductive tract development, on neonatal, prepubertal, and postpubertal prostate gland function of male offspring.

**Methods:**

Fetal rats were exposed to vinclozolin (100 mg/kg body weight) or vehicle control (2.5 mL/kg body weight) *in utero* from gestational day 14 (GD14) to GD19 via oral administration to pregnant dams. Tissue analysis was carried out when male offspring were 0, 4, or 8 weeks of age.

**Results:**

*In utero* exposure to vinclozolin was insufficient to perturb prostatic development and branching, although expression of androgen receptor and mesenchymal fibroblast growth factor-10 was down-regulated. Prostate histology remained normal until puberty, but 100% of animals displayed prostatitis postpubertally (56 days of age). Prostatic inflammation was associated with phosphorylation and nuclear translocation of nuclear factor-kappa B (NFκB) and postpubertal activation of proinflammatory NFκB-dependent genes, including the chemokine interleukin-8 and the cytokine transforming growth factor-β1. Significantly, inflammation arising from vinclozolin exposure was not associated with the emergence of premalignant lesions, such as prostatic intra-epithelial neoplasia or proliferative inflammatory atrophy, and hence mimics nonbacterial early-onset prostatitis that commonly occurs in young men.

**Conclusions:**

These data are the first to unequivocally implicate EDCs as a causative factor and fill an important knowledge gap on the etiology of prostatitis.

Environmental pollutants and industrial chemicals disrupt—and have the potential to alter—the action of gonadal steroid hormones by virtue of their antiandrogenic or estrogenic properties and, in so doing, effect hormonal balance (Foster 2006; Foster and McIntyre 2002; Kelce and Wilson 1997). During fetal and neonatal life, reproductive tract development is hormonally regulated, and the reproductive tract is in an undifferentiated state, lacking compensatory homeostatic mechanisms to prevent adverse effects of endocrine-disrupting chemicals (EDCs) (Cunha et al. 1992; Prins 1997). Thus, the organizational effects of EDCs on the developing reproductive tract can be permanent and irreversible.

Dissimilar to prostate cancer (PCa) and benign prostate hyperplasia (BPH), which predominantly affect aging men, prostate inflammation (prostatitis) affects 9% of men of all ages (McNaughton-Collins et al. 2007). Most (> 90%) prostatitis cases are ascribed to unknown (nonbacterial) origins, and the symptoms, both acute and chronic, are common, bothersome, and burdensome in terms of health-related quality of life (McNaughton Collins et al. 2001; Turner et al. 2005). The economic impact of prostatitis includes an estimated annual expenditure in the United States of > $84 million for diagnosis and management, excluding subsequent pharmaceutical costs (Calhoun et al. 2004; Litwin and Saigal 2007; McNaughton-Collins et al. 2007). Because there are extensive gaps in our understanding of prostatitis etiology, many of these current expenditures may be ineffective and a waste of resources. Thus, it is imperative that we better understand this disease, one that has received relatively little attention compared with BPH and PCa.

Although increased levels of developmental or environmental estrogens have been linked to the increased incidence of prostate disease (Coffey 2001; Harkonen and Makela 2004), chemicals with antiandrogenic activity are potentially of greater importance because androgens are critical to establishing the male phenotype. Vinclozolin [3-(3,5-dichloro-phenyl)-5-methyl-oxazolidine-2,4-dione] is an antiandrogenic systemic dicarboximide fungicide used widely throughout Europe and the United States to control diseases caused by *Botrytis cinerea*, *Sclerotinia sclerotiorum*, and *Moniliniam* spp. Vinclozolin is degraded to the metabolites 2-[(3,5-dichlorophenyl)-carbamoyl]oxy-2-methyl-3-butenoic acid (M1) and 3′,5′-dichloro-2-hydroxy-2-methyl-but-3-enanilide (M2), which are competitive antagonists of androgen receptor (AR) ligand binding, rather than 5α-reductase enzyme inhibitors (Kelce et al. 1994; Wong et al. 1995). When sprayed as Ronilan (a 50% mixture of vinclozolin; BASF AG, Research Triangle Park, NC, USA) on soil, vinclozolin has a half-life of 23 days (Szeto et al. 1989). Previous reports show that vinclozolin exposure in rodents during reproductive tract development induces malformations such as cryptorchidism, hypospadias, and Leydig cell hyperplasia, and permanent changes in sexually dimorphic structures, such as anogenital distance (AGD) and areola/nipple retention (Gray et al. 1994). These effects occur before formation of the hypothalamic–pituitary–gonadal axis and long after vinclozolin has been cleared from the pup; thus, these effects are organizational rather than due to interruption of a feedback loop via the pituitary.

Recent interest in vinclozolin arose from a report that transient embryonic exposure in the rat during embryonic gonadal sex determination [gestation days (GD) 8–14] appears to alter the male germline epigenome and subsequently promotes transgenerational adult-onset disease, including testis and immune abnormalities, prostate and kidney disease, and tumor development (Anway et al. 2005). In a preliminary report, Anway et al. (2006) stated that prostate disease, including inflammation and epithelial atrophy, occurred in aged rats (12–14 months of age) prenatally exposed to vinclozolin, although the incidence of prostatic lesions across four generations of male rats was only 10%. Although the low incidence of prostatic lesions is not compelling, at the same time these findings were controversial because of the vinclozolin purity and the timing and route of its administration *in utero*.

The purity of vinclozolin was not demonstrated by Anway et al. (2006), and vinclozolin, when purchased commercially, requires purification and recrystallization to obtain > 99% purity to ensure that effects are not caused by contaminants. Human exposure to vinclozolin occurs by oral ingestion, enabling metabolism to the more potent AR antagonists (M1 and M2). Direct intraperitoneal administration runs the risk of producing effects not observed by the conventional oral route, such as uterine irritation and changes in uterine blood flow. The timing of vinclozolin exposure also varies the effect on male reproductive tract development in rodents. A window of sensitivity for prostate development occurs when ARs are activated between GD14 and GD19, rather than during embryonic gonadal sex determination around GD8–GD14 (Wolf et al. 2000). Commonly, the outcomes of any transient *in utero* treatments are examined in aging animals. However, antiandrogenic effects also manifest at other times, including pre- and postpuberty, when hormone action is critical for normal prostate maturation and function. Altogether, these variations in treatment protocol may account for the low incidence of prostatic lesions reported by Anway et al. (2005), who used intraperitoneally administered unpurified vinclozolin during GD8–GD14 and did not study outcomes until 12–14 months of age.

Therefore, the aim of this study was to evaluate effects of fetal exposure to purified vinclozolin, administered orally to pregnant rats during the period of male reproductive tract development (GD14–GD19), on pre-and postpubertal prostate gland function in male offspring.

## Material and Methods

### Animals

We conducted all animal procedures according to National Health and Medical Research Council guidelines and the animal experimentation ethics committee at Monash Medical Centre (MMCA/2006/22). Animals were treated humanely and with regard for alleviation of suffering. Time-mated female outbred Sprague-Dawley rats were obtained from Monash University Central Animal Services on GD8 and housed at Monash Medical Centre Animal House under controlled 12-hr light/dark cycle and temperature conditions. Animals were fed *ad libitum*. GD0 was the day vaginal plugs were observed in mated females. Dams and offspring were housed together until weaning [postnatal day (PND) 21], when male litter mates were group-housed, no more than four per cage. Dams and female offspring were euthanized humanely by CO_2_ asphyxiation and were not subjected to postmortem examination.

### Vinclozolin

We obtained vinclozolin from BASF AG (Research Triangle Park, NC, USA) as Ronilan EG (a 50% vinclozolin mixture). We purified and recrystallized the compound. Catalogued as lot no. 357-141A, it was certified as being of > 99% purity by ChemService Inc. (West Chester, PA, USA).

### Treatment

We performed the treatment regime as previously described (Wolf et al. 2000). On GD14, dams were weighed and allocated to treatment groups by body weight (bw) randomization to ensure unbiased weight distribution among groups. Dams were assigned to one of two treatment groups (0 and 100 mg vinclozolin per kilogram bw; *n* = 16 dams per group) and one of three time points of collection [PND0 (*n* = 8), PND28 (*n* = 4), and PND56 (*n* = 4). Dams were orally dosed daily at 1000 hours on GD14–GD19 with 100 mg/kg/bw vinclozolin or corn oil vehicle (2.5 mL/kg/bw; Sigma Chemical Co., St. Louis, MO, USA) and examined for clinical signs of toxicity. The dose chosen corresponds to a level commonly used to investigate vinclozolin effects on male reproductive tract formation, inducing an array of male reproductive tract malformations at high incidence without maternal toxicity. Because the pubertal period in the rodent is controversial, puberty has been defined in relation to functional puberty or the time at which sperm appear and serum testosterone rises. This occurs around PND43 in rats (Korenbrot et al. 1977; Stoker et al. 2000).

### Necropsy of male littermates

Male offspring were collected at PND0, PND28, or PND56; each animal was weighed and euthanized by decapitation (PND0) or CO_2_ asphyxiation (PND28 and PND56), and blood was collected by cardiac puncture for hormonal analysis. External genitalia, including scrotum, prepuce, and penis, were visually examined, and AGD was measured with a caliper.

### Hormone analysis

Serum testosterone levels were measured by ANZAC Research Institute (Sydney, Australia) as previously described (Ly et al. 2001).

### Tissue collection

Using a dissecting microscope (SZX12, Olympus Corp., Tokyo, Japan) and dissecting tools, testes, seminal vesicles (SV), and ventral (VP), dorsal (DP), lateral (LP), and anterior (AP) prostate lobes were dissected from PND28 and PND56 animals and wet weights were recorded. For isolation of PND0 prostates, urogenital tracts were removed and VPs microdissected in a modified watch glass (Maximov depression slide; Fisher, Pittsburgh, PA, USA) in the presence of dissecting media [basal medium of Dulbecco’s modified Eagle’s medium and Hams F-12 (1:1 vol/vol) supplemented with penicillin and streptomycin (5 mL/L) and fungizome (20 μg/mL) at pH 7.3]. Pair-matched organs were fixed in Bouin’s fixative or immediately frozen in liquid nitrogen and stored at –80°C.

### Tissue separation

PND0 VPs for epithelial and mesenchymal RNA analysis were digested in 1% trypsin (Difco, Detroit, MI, USA) in Hank’s calcium- and magnesium-free balanced salt solution (Gibco, Invitrogen, Mount Waverley, Victoria, Australia) for 60 min. Mesenchyme and epithelia were mechanically separated, immediately frozen in liquid nitrogen, and stored at –80°C.

### Histology

Fixed tissues were dehydrated, processed, and embedded in paraffin. Serial 5-μm sections were cut and mounted onto Superfrost Plus+ coated slides (Menzel-Glaser, Braunschweig, Germany). Tissue sections were stained with Harris’s hematoxylin and eosin (H&E) or used for immunohistochemisty. Immunohistochemistry was performed using the DAKO Autostainer Universal Staining System (DAKO A/S, Glostrup, Denmark) (Balanathan et al. 2004). We purchased the following antibodies from Santa Cruz Biotechnology Inc. (Santa Cruz, CA, USA): AR (N-20), transforming growth factor-β1 (TGF-β1; SC-146), toll-like receptor-4 (TLR4), and fibroblast growth factor 10 (Fgf10; H-121:SC_7917). We obtained proliferating cell nuclear antigen (PCNA; clone PC10) from DAKO; CD68 (ED1) from Sapphire Bioscience Pty Ltd. (Redfern, New South Wales, Australia); and phospho-NFκB p65 (Ser536) from Cell Signaling Technology Inc. (Danvers, MA, USA). Antibodies were used as previously described (Bianco et al. 2002; Laczkó et al. 2004; Wang et al. 2001) or according to manufacturer specifications.

### mRNA extraction

Total RNA was extracted from prostate tissues using TRIzol reagent (Invitrogen Life Technologies, Rockville, MD, USA) according to manufacturer specifications and as previously described (Balanathan et al. 2004).

### Oligo gene expression analysis

We conducted gene expression analysis using GEArray DNA microarray (for PND56, array ERN-011.2; for PND0, array EMM-014; SuperArray Bioscience Corp., Frederick, MD, USA) according to manufacturer’s directions. Analysis was conducted on a minimum of four samples per group in duplicate. Briefly, cDNA was synthesized from pure RNA using the TrueLabelling-AMP 2.0 kit (SuperArray Bioscience). cDNA was amplified followed by a 24-hr cRNA synthesis reaction. cRNA concentration and purity were determined by ultraviolet spectrophotometry. After generation and purification of cRNA, we performed array hybridization using the Oligo GEArray HybPlate Basic Protocol (SuperArray Bioscience) according to the manufacturer’s directions. Briefly, arrays were subjected to prehybridization before hybridization with the labeled target cRNA, incubated for 24 hr at 60°C, and then subject to repeated stringency washes. Chemiluminescence was detected using the Chemiluminescent Detection Kit (SuperArray Bioscience). Briefly, arrays were incubated at room temperature in dilute alkaline phosphatase–streptavidin for 10 min, rinsed in buffer, and incubated with CDP-Star chemiluminescent detection reagent. Images were acquired immediately using X-ray exposure. X-ray images were captured using a scanner and saved as 16-bit TIFF images. We completed data analysis using GEArray Expression Analysis Suite (SuperArray Bioscience), with expression normalized to a specific set of housekeeping genes [ribosomal protein L32 (*Rpl32*), lactate dehydrogenase A (*Ldha*), glyceraldehyde 3-phosphate dehydrogenase (*Gadph*), peptidylprolyl isomerase A (*Ppia*)].

### Stereology

We obtained an unbiased estimate of the terminally differentiated secretory epithelial cell population and incidence of inflammation using stereological techniques, based on the Cavalieri principle (Bertram 1995) and as previously described in the testes and prostate (McPherson et al. 2001; Meachem et al. 1996; Singh et al. 1999). Stereological analysis was performed using a BX-51 microscope (Olympus) and a JVC TK-C1380 video camera (Victor Company of Japan Ltd., Yokohama, Japan) coupled to a computer. Images were projected directly onto a video screen; using CAST version 1.10 software (Computer Assisted Stereological Toolbox; Olympus Danmark A/S, Ballerup, Denmark), tissue sections were mapped at 40× magnification to define tissue boundaries. Beginning from a random point, sampling was conducted at predetermined intervals along *x*-and *y*-axes using a 3 × 3-point grid counting frame. We used a minimum of 10 sections per animal, uniformly spaced throughout explants, and five animals per group. To accurately differentiate terminally differentiated secretory epithelial cells, we used tissue sections stained for high-molecular-weight cytokeratin (CKHMW); positively and negatively stained CKHMW cells were identified and percentages of positive and negative cells determined. To determine the incidence of inflammatory lesions, random fields were designated as positive or negative for inflammation. Abnormal inflammatory regions were classified as areas that displayed chronic inflammation.

### Wholemount immunolabeling

We conducted branching morphogenesis analysis on PND0 VPs. Individual VPs were placed in methanol and stored at –20°C. Immunolabeling was performed as previously described by Almahbobi and Hall (1993). Briefly, tissues were permeabilized in 0.2% (vol/vol) Triton X-100 (Sigma) and 5 μg/mL sodium borohydride in phosphate-buffered saline (pH 8.0) for 15 min. Nonspecific binding was blocked with Superblock blocking buffer (Pierce, Rockford, IL, USA) for 1 hr at room temperature, before overnight incubation at 4°C with a monoclonal mouse anti-human CKHMW (5 μg/mL IgG; Dako Corp., Carpinteria, CA, USA). Tissues were incubated with 5 μg/mL goat anti-mouse IgG secondary antibody (F[ab]_2_ fragments) conjugated with fluorescein isothiocyanate (Dako Corp.) for 90 min at room temperature. VPs were mounted on slides using Vectorshield fluorescent mounting medium (Vector Labs, Burlingame, CA, USA) with coverslips mounted on nail polish platforms, to maintain three-dimensional patterns.

### Analysis of branching morphogenesis

We generated serial optical images of CKHMW-stained whole-mount tissues at 2 μm interframe steps using an Olympus confocal microscope, and captured and stored them in 8-bit BMP format using Fluroview software (Olympus) as previously described (Almahbobi et al. 2005). Confocal images were subsequently used to construct a three-dimensional skeleton representing the original ductal pattern of the gland, using lines running through the center of each ductal/epithelial branch. A full description of this process was previously reported for the study of branching morphogenesis in kidney (Cullen-McEwen et al. 2002; Fricout et al. 2001) and prostate (Almahbobi et al. 2005). The resultant algorithm provides fully automated measurements of the branch length (in pixels and micrometers) and cumulative surface areas (in square pixels) of the individual ducts as they appear in all the frames. Total ductal length was calculated by adding together individual branch lengths from multiple ducts within a lobe. The value of the surface areas was multiplied by 1.5522 (pixel area) to convert it into micrometers, and then by 2 (for 2-μm interframe steps) to obtain total volume (in cubic micrometers) of individual ducts. Numbers of branch points, branches, and terminal tips were automatically generated by the software.

### Statistical analysis

All pup data was analyzed individually and nested by dam to yield litter means. To test for significance of treatment effects, we corrected for litter as a main effect variable using one-way analysis of covariance on SPSS, version 16 (SPSS Inc., Chicago, IL, USA); data are expressed as litter mean ± SE. AGD and organ weights were analyzed with body weight as a covariate. Control and vinclozolin were compared using an *F*-test, with the significance threshold at 5% (*p* < 0.05). Analysis of control and vinclozolin stereological data was performed using a two-tailed paired *t*-test using Prism 4.0 software (GraphPad Software, Inc., San Diego, CA, USA).

## Results

### PND0 litter data

We compared pregnant Sprague-Dawley rats transiently exposed to vinclozolin (100 mg/kg/day) on GD14–GD19 with corn oil–treated controls (2.5 mL/kg/day). Vinclozolin did not induce maternal toxicity or affect normal pregnancy: no dams presented dystocia or delivered late. Dam weight gain through the dosing period was not significantly different between groups (Table 1). Vinclozolin treatment did not affect live litter size or sex ratio, with sex confirmed in offspring at puberty (Table 1).

### Gross analysis of male offspring after in utero treatment

We analyzed male offspring exposed *in utero* on PND0 (day of birth), PND28 (prepubertal), and PND56 (post-pubertal) for any weight differences or gross morphologic abnormalities. Pup weights on PND0 were significantly lower in the vinclozolin-treated group compared with the control group (*p* < 0.05) (Table 1), but no significant changes in body weight were observed on PND28 or PND56 (*p* < 0.05; Table 2). AGD, a sensitive indicator of antiandrogenicity, was significantly reduced at all ages in the vinclozolin group compared with controls (Tables 1 and 2). Covariate analysis demonstrated that the PND0 AGD reductions were not due to pup weight reductions.

**Figure 1:**
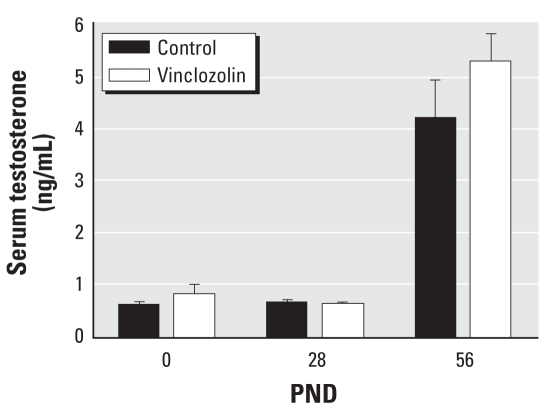
Effects of 6-day exposure to vinclozolin or corn oil (control) on serum testosterone (mean ± SE) on PND0, PND28, or PND56.

On PND28, compared with controls, vinclozolin treatment did not significantly reduce testis, SV, VP, AP, DP, or LP weights (*p* < 0.05) (Table 2). Analysis of external genitalia revealed undescended testes in 35.93 ± 4.37% of prepubertal animals exposed to vinclozolin, compared with none in the control group. At PND56, vinclozolin treatment significantly reduced SV and VP weights and significantly increased AP weight (*p* < 0.05) (Table 2). We observed no significant differences in LP, DP, and testis weights (Table 2). Malformations of external genitalia included cleft prepuce, incomplete preputial separation, cleft phallus, and hypospadias, observed in 47.02 ± 7.92% of postpubertal animals exposed to vinclozolin compared with none in controls. Analysis of serum testosterone levels revealed no significant differences between control and vinclozolin groups at any postnatal age (Figure 1).

Although we collected and analyzed all prostate lobes, we report here only data for the VP because, in addition to being the most commonly reported lobe with respect to EDC exposures, it is the most androgen-sensitive lobe (Prins and Birch 1995; Risbridger et al. 2001); therefore, the actions of an anti-androgenic chemical are more likely to induce effects in this lobe.

### Absence of perturbations in branching morphogenesis in neonatal offspring

The inductive and instructive potential of the mesenchyme and importance of mesenchymal androgen signaling in normal prostate branching and development have been well established. To examine whether *in utero* vinclozolin exposure perturbed normal mesenchymal signaling in offspring, we performed gene array analysis of 113 common growth factors on neonatal mesenchyme. Significant (> 1.5-fold) down-regulation occurred for several mesenchymal genes, including *Fgf10*, in prostates from animals exposed *in utero* to vinclozolin compared with controls; this was confirmed by immunoprotein localization (data not shown). Because estrogen exposure down-regulates *Fgf10* and perturbs normal prostate ductal branching (Huang et al. 2005), we investigated whether the antiandrogen-induced reduction in mesenchymal *Fgf10* was associated with developmental abnormalities.

**Figure 2:**
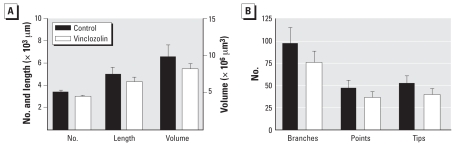
Effects of *in utero* exposure to corn oil (control) or vinclozolin on branching morphogenesis in the prostate of PND0 males. Neither ductal number, length, and volume (*A*) nor number of branches, points, and tips (*B*) revealed significant differences between treatment groups. Values shown are mean ± SE.

We analyzed branching morphogenesis in neonatal tissues using a computer-based method that allows examination of temporal and spatial alterations in branching morphogenesis as a result of experimental manipulations. Despite mesenchymal *Fgf10* reductions, prostate size was normal, with no significant differences present in ductal number, length, volume, or branch points in neonatal specimens between prostates from animals in the vinclozolin group compared with controls (Figure 2).

### Normal prostate development until puberty with early onset of prostate inflammation in postpubertal offspring

**Figure 3:**
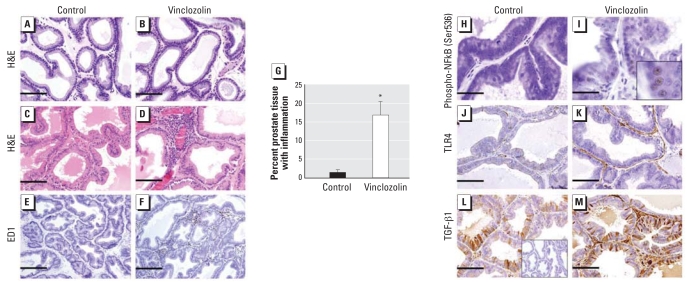
Effect of *in utero* exposure to vinclozolin on early-onset postpubertal prostatitis. (*A*–*F*) Photomicrographs showing morphology of prostates from control (*A,C,E*) and vinclozin-exposed (*B,D,F*) rats. Prepubertally, no morphologic differences were identified between prostates in the two groups (*A,B*). Prostates from controls (*C*) showed extensive ductal branching and canalization, with pseudostratified columnar epithelial cells lining the ducts and continuous stromal sheaths surrounding the ducts; however, in prostates from vinclozolin-exposed rats (*D*), prominent but focal regions of inflammation are evident. Compared with controls (*E*), an increase in the proportion of macrophages surrounding the ducts and infiltrating into vessels is evident after vinclozolin treatment (*F*). (*G*) Percentage of inflammation (mean ± SE) in prostates of control and vinclozolin-exposed rats. (*H*–*M*) Photomicrographs of prostates from control (*H,J,L*) and vinclozin-exposed (*I,K,M*) rats immunostained for phospho-NFκB p65 (Ser536) (*H,I*), TLR-4 (*J,K*), and TGF-β1 (*L,M*). Compared with control tissues (*H*), activation of the inflammatory NFκB pathway was evident in prostates from vinclozolin-treated animals, with increased nuclear immunoprotein localization of phospho-NFκB p65 (Ser536) (*I* and inset). (*J*–*M*) Compared with control prostates (*J*), vinclozolin-exposed prostates showed NFκB-dependent up-regulated expression of the *Tlr4* gene (*K*) and an increase in TGF-β1 expression (*L*, *M*). The inset in *L* is an IgG-matched negative control. Bars = 50 μm in *A–D*, *H*, *I*, *L*, and *M*; 20 μm in *E* and *F*; and 100 μm in *J*, and *K*. *p < 0.05.

We observed no gross morphologic differences between *in utero* vinclozolin and control treatment in PND28 (prepubertal) prostates (Figure 3A,B). However, analysis of prostate specimens at PND56 (postpubertal) revealed the onset of prostate inflammation in *in utero* vinclozolin-exposed males (Figure 3C,D). We observed prominent, but focal, regions of inflammation in 100% of animals, with an increase in the proportion of inflammatory cells, particularly leukocytes and macrophages, surrounding the ducts and infiltrating the vessels (Figure 3D). Increased macrophage infiltration was evident in prostates from animals exposed to vinclozolin, as demonstrated by immunolocalization of ED1 (Figure 3E,F), but was absent in control animals. *In utero* vinclozolin treatment resulted in a significant increase (*p* < 0.05) in the percentage of prostatic inflammatory lesions, identified by stereological analysis, from 1.4 ± 0.80% to 16.8 ± 3.72% (Figure 3G).

Proinflammatory stimuli and immune responses are commonly controlled by the nuclear factor-kappa B (NFκB) family of transcription factors. In unstimulated cells, NFκB is sequestered in the cytoplasm of cells and activated when it is phosphorylated and translocated to the nucleus. We confirmed activation of NFκB in prostates exposed *in utero* to vinclozolin by nuclear immunoprotein localization of phospho-NFκB p65 (Ser536) antibody, which detects NFκB p65 only when phosphorylated at serine 536. In control prostates, we identified few immunopositive cells (Figure 3H,I).

Activation of NFκB subsequently induces transcription of many NFκB-dependent genes, including those encoding inflammatory cytokines and chemokines. To determine the distinct pattern of gene expression after activation of NFκB signaling by vinclozolin treatment, we performed pathway-specific gene array analysis of 113 key genes involved in the inflammatory response. We observed significant (> 1.5-fold) up- and down-regulation of 69 and 34 genes, respectively, in prostate tissues of vinclozolin-exposed PND56 animals compared with controls (Table 3). These data show increased activation of classic proinflammatory NFκB-dependent genes, including chemokines such as interleukin-1α (IL-1α), IL-6, and IL-8 and cytokines such as TGF-β and tumor necrosis factor-α (TNF-α), as well as other ligands and receptors, including TLR1–TLR6, TLR9, and TNF receptors.

We selected several key NFκB-dependent genes to examine transcriptional activity by immunoprotein localization, including TLR4 and TGF-β1. We confirmed heightened expression of TLR4, an important innate immune receptor, by increased immunoprotein localization, particularly in the stromal and periductal compartments of tissues from vinclozolin-exposed animals (Figure 3J,K). We observed a significant up-regulation of TGF-β1 expression in tissues from the vinclozolin group (Figure 3L,M), correlating with a significant down-regulation of the immunosuppressive cytokine and TGF-β1–negative regulator IL-10 (Table 3).

### Epithelial aberrations in postpubertal offspring without evidence of premalignancy

**Figure 4:**
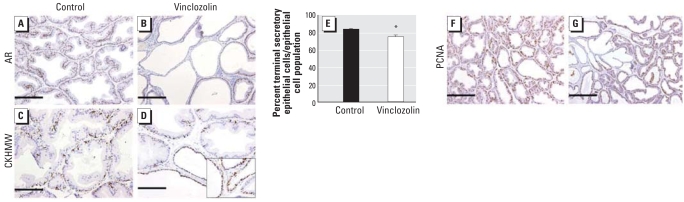
Epithelial attenuation in postpubertal animals after *in utero* corn oil (control) or vinclozolin treatment. (*A–D*) Photomicrographs of prostates from control (*A,C*) and vinclozin-exposed (*B,D*) rats showing immunolocalization of AR (*A,B*) and CKHMW (*C,D*). In tissues from controls (*A*), AR is localized predominately to epithelial cells, but a down-regulation in epithelial AR expression is observed in vinclozolin-exposed prostates (*B*). Immunolocalization for CKHMW indicated a discontinuous layer of basal cells in control tissues (*C*) compared with a continuous layer in prostates from vinclozolin-exposed animals (*D*). (*E*) Stereologic analysis showing a significant loss of terminally differentiated epithelial cells in vinclozolin-exposed animals compared with controls; data are mean ± SE. (*F*,*G*) Proliferative activity examined by immunolocalization of PCNA. A reduction in immunopositive epithelial cells was observed in attenuated glands of prostate tissues from vinclozolin-exposed animals (*G*) compared with controls (*F*). Bars = 100 μm in *A* and *B*; 50 μm in *C* and *D*; and 200 μm in *F* and *G*. *p < 0.05.

In addition to the 100% penetrance of prostatic inflammation observed in PND56 prostate specimens, *in utero* exposure to vinclozolin also induced focal epithelial attenuation (reduction in epithelial cell height and thinning of ductal structure) in all animals. We observed a reduction in epithelial AR concurrently with a reduction in terminally differentiated secretory epithelia (Figure 4A,B). Immunolocalization of the basal cell marker CKHMW (Figure 4C,D) showed loss of terminally differentiated secretory epithelia and epithelial attenuation, evidenced by a continuous layer of basal cells compared with a discontinuous layer observed in control, confirmed by stereologic analysis (Figure 4E).

Prostatic inflammation associated with atrophy and proliferation [known as proliferative inflammatory atrophy (PIA)] has been reported as a premalignant lesion in men. Although PIA has not been confirmed in rodents, we examined proliferative activity by immunolocalization of PCNA (Figure 4F,G). Epithelial attenuated glands of tissues exposed *in utero* to vinclozolin showed an apparent loss of proliferative activity with a reduction in immunopositive epithelial cells, demonstrating the absence of pathology comparable with PIA in these tissues. There was no evidence of other prostatic lesions, such as premalignant prostatic intraepithelial neoplastic lesions.

## Discussion

The role of EDCs in the early origins of adult prostate disease is of concern and controversy to the lay and scientific communities. These data are the first to unequivocally implicate the antiandrogenic activity of EDCs as causative factors in the etiology of prostatitis in the rat, providing novel insight to the origins of this disease for which > 90% of human cases are of unknown cause (McNaughton-Collins et al. 2007).

The longer-term consequences of *in utero* vinclozolin exposure include the development of gross malformations of the male reproductive tract, such as the epididymis, vas deferens, SVs, prostate, external genitalia (hypospadias), cryptorchidism and testicular injury, and permanent change in sexually dimorphic structures (Wolf et al. 2000). A reduction in nuclear epithelial AR in prostate tissues from animals exposed *in utero* to vinclozolin correlates with previous studies demonstrating rapid AR degradation after antiandrogen binding (Wong et al. 1995).

The immediate effects of reduced AR and mesenchymal *Fgf10* expression in tissues exposed *in utero* to vinclozolin do not result in any perturbations in prostate branching. This implies that other androgen-regulated paracrine factors produced by the mesenchyme were sufficient to compensate and induce normal differentiation and development. Furthermore, the absence of a branching effect after *in utero* treatment during primary gland genesis may indicate that *Fgf10* acts only with concurrent chemical exposure in the neonate when secondary branching morphogenesis occurs. These findings are in contrast with effects reported after neonatal estrogen exposure, in which reductions in mesenchymal *Fgf10* inhibit branching morphogenesis (Huang et al. 2005).

Prostatic inflammation is a common feature of endocrine disruption by estrogenic and antiandrogenic chemicals (Naslund et al. 1988; Prins 1997; Stoker et al. 1999). Results of the present study demonstrate the absence of any morphologic changes before puberty but an inflammatory response in all young post-pubertal (PND56) prostates after *in utero* anti-androgenic exposure. Activation of the NFκB inflammatory pathway was evident, with a significant down-regulation of AR expression. There is considerable evidence to show crosstalk between AR and NFκB (De Bosscher et al. 2006); thus, it is reasonable to postulate that the persistent repression of AR signaling induced by vinclozolin results in androgenic activity that is insufficient to suppress NFκB signaling pathways, resulting in inappropriate activation of NFκB and the emergence of prostatitis. Although the exact mechanism by which estradiol exposure promotes an inflammatory response in the adult prostate has not yet been determined, antiandrogen-induced inflammation is associated with activation of the “canonical” proinflammatory NFκB inflammatory signaling pathway and NFκB-dependent genes.

There are further important differences between EDCs that are antiandrogenic or estrogenic. In contrast with estrogen-induced inflammation, the long-term effects of transient *in utero* exposure to vinclozolin failed to induce premalignancy. Inflammation and focal atrophy associated with increased proliferation have been described in human prostate specimens as PIA and may be premalignant. The pathology arising from *in utero* vinclozolin treatment and described herein does show inflammation and focal epithelial attenuation but in the absence of increased proliferation. Therefore, we conclude that vinclozolin did not induce the premalignant lesion PIA.

## Conclusions

Overall, the robust incidence of inflammation in 100% of young adult rats more closely mimics human nonbacterial prostatitis that occurs in young men. Ninety percent of prostatitis cases are of unknown cause, and these data are the first to implicate antiandrogenic EDCs as a causative factor in the etiology of this inflammatory disease of the prostate via activation of the classical NFκB inflammatory pathway. Although the level of vinclozolin used in this study far exceeds that observed in the environment and projected human exposure, these results raise further concerns that *in utero* exposures to EDCs with antiandrogenic activity have long-range effects that include the development of prostatitis in early adult life. Our results also provide further impetus to test the efficacy of treatments that block or abrogate NFκB signaling in the treatment of prostatitis.

## Figures and Tables

**Table 1:** Effects of 6-day exposure to corn oil (control) or vinclozolin on PND0 in dams and pups.

End point	Control	Vinclozolin
Dams assigned	16	16
Dams pregnant	12	13
Dams delivered late	0	0
Dam weight gain through dosing period (g)	49.29 ± 10.38	53.33 ± 11.81
Live litter size	12.18 ± 2.26	12.83 ± 2.33
Male:female ratio at birth	1.10 ± 0.67	1.61 ± 1.80
Pup weight at birth (g)	6.44 ± 0.07	6.16 ± 0.09*
AGD in male offspring at birth	4.36 ± 0.08	3.75 ± 0.10*

Values shown are number or mean ± SE.

* *p* < 0.05.

**Table 2:** Effects of 6-day exposure to corn oil (control) or vinclozolin on reproductive organ weights (litter mean ± SE) in male offspring on PND28 and PND56.

	Control		Vinclozolin	
End point	PND28	PND56	PND28	PND56
Body weight (g)	70.97 ± 0.61	259.09 ± 7.21	73.6 ± 1.25	275.29 ± 5.02
AGD (mm)	25.55 ± 0.54	40.51 ± 0.91	24.20 ± 0.33*	30.82 ± 1.31*
VP weight (mg)	37.67 ± 1.45	223.74 ± 6.81	35.38 ± 1.25	199.16 ± 6.09*
AP weight (mg)	4.56 ± 0.55	69.32 ± 3.67	4.27 ± 0.32	82.23 ± 3.23*
LP weight (mg)	7.3 ± 0.49	45.68 ± 4.04	5.73 ± 0.61	56.69 ± 3.63
DP weight (mg)	9.1 ± 1.23	61.26 ± 4.42	9.15 ± 0.71	73.25 ± 3.98
SV weight (mg)	11.61 ± 0.96	507.94 ± 15.14	12.96 ± 0.58	373.15 ± 13.56*
Testis weight (g)	0.525 ± 0.02	2.41 ± 0.06	0.484 ± 0.01	2.58 ± 0.05

* *p* < 0.05.

**Table 3:** Effects of 6 day *in utero* exposure to vinclozolin on key NFκB-dependent inflammatory genes on PND56 compared with corn oil (control) treatment.

UniGene^a^	Symbol	Description	Fold difference
Rn.12300	*Il1a*	Interleukin 1 alpha	5.05
Rn.9869	*Il1b*	Interleukin 1 beta	2.02
Rn.1716	*Il6ra*	Interleukin 6 receptor, alpha	5.16
Rn.12138	*Il6st*	Interleukin 6 signal transducer	11.13
Rn.138115	*Il8ra*	Interleukin 8 receptor, alpha	7.80
Rn.90347	*Il8rb*	Interleukin 8 receptor, beta	4.29
Rn.92374	*Il9*	Interleukin 9	3.52
Rn.10045	*Il9r*	Interleukin 9 receptor	1.96
Rn.54465	*Itgam*	Integrin alpha M	3.34
N/A	*LOC301289*	Similar to interleukin-17 precursor (IL-17) (cytotoxic T lymphocyte-associated antigen 8) (CTLA-8)	13.43
Rn.2661	*Mif*	Macrophage migration inhibitory factor	9.19
Rn.10400	*Nos2*	Nitric oxide synthase 2, inducible	19.35
Rn.29157	*Rac1*	Ras-related C3 botulinum toxin substrate 1	9.89
Rn.40136	*Tgfb1*	Transforming growth factor, beta 1	27.30
Rn.107212	*Tlr1*	Similar to toll-like receptor 1 (LOC305354), mRNA	18.47
Rn.46387	*Tlr2*	Toll-like receptor 2	27.23
Rn.15273	*Tlr3*	Toll-like receptor 3	31.22
Rn.14534	*Tlr4*	Toll-like receptor 4	14.04
Rn.198962	*Tlr5*	Toll-like receptor 5	14.86
Rn.163249	*Tlr6*	Toll-like receptor 6	14.42
Rn.92495	*Tlr9*	Toll-like receptor 9	22.90
Rn.2275	*Tnf*	Tumor necrosis factor (TNF superfamily, member 2)	27.49
Rn.11119	*Tnfrsf1a*	Tumor necrosis factor receptor superfamily, member 1a	23.49
Rn.83633	*Tnfrsf1b*	Tumor necrosis factor receptor superfamily, member 1b	29.62
Rn.30043	*Tnfsf4*	Tumor necrosis factor (ligand) superfamily, member 4	16.82
Rn.44218	*Cd40lg*	CD40 ligand	9.51
Rn.9868	*Il10*	Interleukin 10	0.44
Rn.50003	*Il17b*	Interleukin 17B	0.79
Rn.11118	*Il18*	Interleukin 18	0.92

a Data from National Center for Biotechnology Information (2008).

## References

[b1-ehp0116-000923] Almahbobi G, Hall PF (1993). Indirect immunofluorescence modified to display two antigens with one light filter. Histochem J.

[b2-ehp0116-000923] Almahbobi G, Hedwards S, Fricout G, Jeulin D, Bertram JF, Risbridger GP (2005). Computer-based detection of neonatal changes to branching morphogenesis reveals different mechanisms of and predicts prostate enlargement in mice haplo-insufficient for bone morphogenetic protein 4. J Pathol.

[b3-ehp0116-000923] Anway MD, Cupp AS, Uzumcu M, Skinner MK (2005). Epigenetic transgenerational actions of endocrine disruptors and male fertility. Science.

[b4-ehp0116-000923] Anway MD, Leathers C, Skinner MK (2006). Endocrine disruptor vinclozolin induced epigenetic transgenerational adult-onset disease. Endocrinology.

[b5-ehp0116-000923] Balanathan P, Ball EM, Wang H, Harris SE, Shelling AN, Risbridger GP (2004). Epigenetic regulation of inhibin alpha-subunit gene in prostate cancer cell lines. J Mol Endocrinol.

[b6-ehp0116-000923] Bertram JF (1995). Analyzing renal glomeruli with the new stereology. Int Rev Cytol.

[b7-ehp0116-000923] Bianco JJ, Handelsman DJ, Pedersen JS, Risbridger GP (2002). Direct response of the murine prostate gland and seminal vesicles to estradiol. Endocrinology.

[b8-ehp0116-000923] Calhoun EA, McNaughton Collins M, Pontari MA, O’Leary M, Leiby BE, Landis RJ (2004). The economic impact of chronic prostatitis. Arch Intern Med.

[b9-ehp0116-000923] Coffey DS (2001). Similarities of prostate and breast cancer: evolution, diet, and estrogens. Urology.

[b10-ehp0116-000923] Cullen-McEwen LA, Fricout G, Harper IS, Jeulin D, Bertram JF (2002). Quantitation of 3D ureteric branching morphogenesis in cultured embryonic mouse kidney. Int J Dev Biol.

[b11-ehp0116-000923] Cunha G, Alarid E, Turner T, Donjacour A, Boutin E, Foster B (1992). Normal and abnormal development of the male urogenital tract. Role of androgens, mesenchymal-epithelial interactions, and growth factors. J Androl.

[b12-ehp0116-000923] De Bosscher K, Vanden Berghe W, Haegeman G (2006). Crosstalk between nuclear receptors and nuclear factor kappaB. Oncogene.

[b13-ehp0116-000923] Foster PM (2006). Disruption of reproductive development in male rat offspring following in utero exposure to phthalate esters. Int J Androl.

[b14-ehp0116-000923] Foster PM, McIntyre BS (2002). Endocrine active agents: implications of adverse and non-adverse changes. Toxicol Pathol.

[b15-ehp0116-000923] Fricout G, Cullen-McEwen L, Harper IS, Jeulin D, Bertram JF (2001). A quantitative method for analysing 3D branching in embryonic kidneys: development of a technique and preliminary data. Image Anal Stereol.

[b16-ehp0116-000923] Gray LE, Ostby JS, Kelce WR (1994). Developmental effects of an environmental antiandrogen: the fungicide vinclozolin alters sex differentiation of the male rat. Toxicol Appl Pharmacol.

[b17-ehp0116-000923] Harkonen PL, Makela SI (2004). Role of estrogens in development of prostate cancer. J Steroid Biochem Mol Biol.

[b18-ehp0116-000923] Huang L, Pu Y, Alam S, Birch L, Prins GS (2005). The role of Fgf10 signaling in branching morphogenesis and gene expression of the rat prostate gland: lobe-specific suppression by neonatal estrogens. Dev Biol.

[b19-ehp0116-000923] Kelce WR, Monosson E, Gamcsik MP, Laws SC, Gray LE (1994). Environmental hormone disruptors: evidence that vinclozolin developmental toxicity is mediated by antiandrogenic metabolites. Toxicol Appl Pharmacol.

[b20-ehp0116-000923] Kelce WR, Wilson EM (1997). Environmental antiandrogens: developmental effects, molecular mechanisms, and clinical implications. J Mol Med.

[b21-ehp0116-000923] Korenbrot CC, Huhtaniemi IT, Weiner RI (1977). Preputial separation as an external sign of pubertal development in the male rat. Biol Reprod.

[b22-ehp0116-000923] Laczkó I, Hudson DL, Freeman A, Feneley MR, Masters JR (2004). Comparison of the zones of the human prostate with the seminal vesicle: morphology, immunohistochemistry, and cell kinetics. Prostate.

[b23-ehp0116-000923] Litwin M, Saigal C (2007). Urologic Diseases in America.

[b24-ehp0116-000923] Ly LP, Jimenez M, Zhuang TN, Celermajer DS, Conway AJ, Handelsman DJ (2001). A double-blind, placebo-controlled, randomized clinical trial of transdermal dihydrotestosterone gel on muscular strength, mobility, and quality of life in older men with partial androgen deficiency. J Clin Endocrinol Metab.

[b25-ehp0116-000923] McNaughton-Collins M, Joyce G, Wise M, Pontari M, Litwin M, Saigal C (2007). Prostatitis. Urologic Diseases in America.

[b26-ehp0116-000923] McNaughton Collins M, Pontari MA, O’Leary MP, Calhoun EA, Santanna J, Landis JR (2001). Quality of life is impaired in men with chronic prostatitis: the Chronic Prostatitis Collaborative Research Network. J Gen Intern Med.

[b27-ehp0116-000923] McPherson S, Wang H, Jones M, Pedersen J, Iismaa T, Wreford N (2001). Elevated androgens and prolactin in aromatase deficient (ArKO) mice cause enlargement but not malignancy of the prostate gland. Endocrinology.

[b28-ehp0116-000923] Meachem SJ, McLachlan RI, de Kretser DM, Robertson DM, Wreford NG (1996). Neonatal exposure of rats to recombinant follicle stimulating hormone increases adult Sertoli and spermatogenic cell numbers. Biol Reprod.

[b29-ehp0116-000923] Naslund MJ, Strandberg JD, Coffey DS (1988). The role of androgens and estrogens in the pathogenesis of experimental nonbacterial prostatitis. J Urol.

[b30-ehp0116-000923] National Center for Biotechnology Information (2008). Unigene.

[b31-ehp0116-000923] Prins G, Naz R (1997). Developmental estrogenization of the prostate gland. Prostate: Basic and Clinical Aspects.

[b32-ehp0116-000923] Prins G, Birch L (1995). The developmental pattern of androgen receptor expression in rat prostate lobes is altered after neonatal exposure to estrogen. Endocrinology.

[b33-ehp0116-000923] Risbridger GP, Wang H, Frydenberg M, Cunha G (2001). The metaplastic effects of estrogen on mouse prostate epithelium: proliferation of cells with basal cell phenotype. Endocrinology.

[b34-ehp0116-000923] Singh J, Zhu Q, Handelsman DJ (1999). Stereological evaluation of mouse prostate development. J Androl.

[b35-ehp0116-000923] Stoker TE, Laws SC, Guidici DL, Cooper RL (2000). The effect of atrazine on puberty in male Wistar rats: an evaluation in the protocol for the assessment of pubertal development and thyroid function. Toxicol Sci.

[b36-ehp0116-000923] Stoker TE, Robinette CL, Cooper RL (1999). Perinatal exposure to estrogenic compounds and the subsequent effects on the prostate of the adult rat: evaluation of inflammation in the ventral and lateral lobes. Reprod Toxicol.

[b37-ehp0116-000923] Szeto SY, Burlinson NE, Rahe JE, Oloffs PC (1989). Kinetics of hydrolysis of the dicarboximide fungicide vinclozolin. J Agric Food Chem.

[b38-ehp0116-000923] Turner JA, Ciol MA, Von Korff M, Berger R (2005). Health concerns of patients with nonbacterial prostatitis/pelvic pain. Arch Intern Med.

[b39-ehp0116-000923] Wang Y, Hayward S, Cao M, Thayer K, Cunha G (2001). Cell differentiation lineage in the prostate. Differentiation.

[b40-ehp0116-000923] Wolf CJ, LeBlanc GA, Ostby JS, Gray LE (2000). Characterization of the period of sensitivity of fetal male sexual development to vinclozolin. Toxicol Sci.

[b41-ehp0116-000923] Wong C, Kelce WR, Sar M, Wilson EM (1995). Androgen receptor antagonist versus agonist activities of the fungicide vinclozolin relative to hydroxyflutamide. J Biol Chem.

